# Sulfated Polysaccharides in Cancer Therapy: A Focus on Algal-Derived Bioactive

**DOI:** 10.3390/md24040131

**Published:** 2026-03-31

**Authors:** N. M. Liyanage, D. S. Dissanayake, Yiqiao Li, Kyung Yuk Ko, D. P. Nagahawatta, You-Jin Jeon

**Affiliations:** 1 Department of Medicine, Faculty of Medicine and Dentistry, University of Alberta, Edmonton, AB T6G 2B7, Canada; 2 Department of Marine Life Sciences, Jeju National University, Jeju 690-756, Republic of Korea

**Keywords:** sulfated polysaccharides, anticancer, metastasis, apoptosis, invasion, nanoparticles, fucoidan

## Abstract

Sulfated polysaccharides (SPs), biologically active macromolecules from marine and terrestrial organisms, hold significant potential in revolutionizing cancer therapy. Characterized by their unique sulfate ester groups and structural diversity, SPs exhibit a broad spectrum of bioactivities, including immunomodulation, apoptosis induction, metastasis suppression, and angiogenesis inhibition. Prominent SPs, such as fucoidan from brown algae and carrageenan from red algae, have shown remarkable anticancer properties, either as standalone agents or in synergy with conventional therapies like chemotherapy and radiotherapy. Their mechanisms of action involve targeting critical pathways such as NF-kB, VEGF, and PI3K/Akt, disrupting cancer cell proliferation, invasion, and tumor microenvironment dynamics. SPs also enhance immune system responses, reduce chemotherapy-induced side effects, and exhibit antioxidant properties, making them versatile candidates in cancer treatment. Innovations like SP-based nanoparticles are addressing bioavailability and drug delivery challenges, providing targeted and sustained therapeutic effects while minimizing off-target toxicity. Despite their promise, challenges such as structural complexity, scalability, and clinical validation hinder their widespread adoption. This review provides a comprehensive analysis of SPs’ therapeutic potential, mechanisms, and emerging applications in oncology. It emphasizes the need for advanced extraction, characterization techniques, and clinical research to unlock their full potential, paving the way for novel, efficient, and safer cancer therapies.

## 1. Introduction

Cancers comprise diseases that occur due to the growth and survival of abnormal cells, and they cause harm to the neighboring tissues by invading them and spreading to the other parts of the body. This has become a serious issue worldwide, with millions of illnesses and deaths each year [1]. Even though there are numerous treatment procedures such as chemotherapy, immunotherapy, surgery, and targeted therapy, successful management of cancers is still challenging. Tumor heterogeneity, resistance to treatments, potential side effects, and limited efficiency of the treatments in advanced-stage patients hinder the better outcomes of available cancer treatments [2].

Sulfated polysaccharides (SPs) are complex carbohydrates that are distinguished by the sulfate ester groups that are present in their sugar residues, and the chemical and biological properties of the SPs vary with them [3]. They are widely distributed, and their functional and ecological adaptability is demonstrated by a variety of sources, such as fungi, mammals, and marine organisms [4]. Carrageenans and fucoidans are SPs from marine red and brown algae that function by providing structural support and protection. The fungal cell wall consists of chitin derivatives and sulfated glucans that provide the protection and integrity of the cell wall [5]. Heparin and chondroitin sulfate are polysaccharides from mammals that are the components of their extracellular matrix, and they help in cell signaling, and tissue repair, and aid in coagulation [6]. Their unique structure and excellent bioactivities are useful in numerous applications such as pharmaceuticals, nutraceuticals, and biotechnology. Among these, fucoidan has gained particular attention due to its distinctive sulfated fucose-rich structure and its wide range of biological activities, including anticancer, anti-inflammatory, and immunomodulatory effects [7]. The increasing number of studies exploring its structure-activity relationship and therapeutic applications further supports its selection as the primary focus of this review.

Having limitations in current cancer treatments showcases the pressing need for new strategies, especially in advanced malignant situations. The development of novel therapeutics including targeted therapies, immunotherapies, and nanotechnology-based interventions, as well as the investigation of novel natural compounds, synthetic drugs, and modern technological approaches may help in safer and more efficient cancer therapies [8].

This review comprehensively elaborates on the structural variability, biological activities, and potential therapeutic effects of SPs in cancer treatment. An analysis of these compounds from various natural sources, highlighting their unique chemical characteristics that aid in cancer therapy is discussed. Furthermore, this review provides insight into their mechanism of action related to cancer therapy, including immunomodulation, metastasis suppression, and angiogenesis inhibition. Also, the challenges and future directions, opportunities, and clinical applications are addressed focusing on scalability and formulation strategies for drug development.

## 2. Structural Characteristics of SPs

The structure of SPs varies greatly depending on their monosaccharide composition, molecular weight, linkage type, and degree of sulfation, and these variations greatly impact the solubility, conformation, and interactions with other molecules, making polysaccharides highly versatile [9].

### 2.1. Structural Variability and Composition

The molecular weight and the chain length of the polysaccharides vary based on their origin and the biosynthetic processes undergone. Higher molecular weight polysaccharides might interact with cellular membranes and proteins in a different manner since they often exhibit greater viscosity. Polysaccharides with shorter chain lengths are better with biological activities since they possess higher solubility and better absorption ability [10]. Molecular weight significantly affects biological performance. High-molecular-weight SPs often exhibit stronger anticoagulant and immunomodulatory effects due to multivalent binding and receptor clustering [11]. In contrast, low-molecular-weight fractions generally demonstrate improved solubility, tissue penetration, and cellular uptake, contributing to enhanced anticancer and anti-inflammatory activities. Thus, molecular weight influences both pharmacokinetics and signaling efficiency [12].

The composition of SPs consists of several sugar monomers, which are considered as the building blocks, and they include Uronic acids such as Glucuronic acid and iduronic acid that contribute to charge properties and structure of polysaccharides; Hexosamines such as Glucosamine, Galactosamine, and derivatives; and neutral sugars such as glucose, fucose, and galactose [13]. Sulfate ester groups are present in sugar residues and attached to the hydroxyl groups. The sulfate groups bound to carbon positions 2, 4 or 6 of the sugar units are the main cause of sulfate polysaccharides’ negative charge and bioactivity [14].

The monosaccharide composition and its specific arrangement in the polysaccharide chain greatly affect the structural diversity. For instance, Carrageenans present in red seaweed consist of galactose units, while fucoidans, found in brown seaweeds, are rich in fucose. These sugar sequences determine the three-dimensional arrangements and interactions of the polysaccharide with other molecules [15]. The type and arrangement of monosaccharide residues determine chain flexibility, sulfate distribution, and molecular conformation. Fucose-rich SPs are commonly associated with anticancer and anti-inflammatory effects, whereas galactose-based SPs often exhibit antiviral activity [16]. Additionally, conformational features such as helical or flexible structures regulate accessibility to binding sites and may enable SPs to mimic endogenous glycosaminoglycans, thereby modulating growth factor signaling pathways [17].

Glycosidic bonds are the type of bonds in which sugar monomers are connected in a sulfated polysaccharide structure. The α or β glycosidic linkage in specific monomer configurations determines the overall structure and function of the polysaccharide [13].

### 2.2. Sulfation Patterns and Degree of Substitution

Sulfation occurs when sulfate groups are introduced onto specific hydroxyl positions of sugar units in the polysaccharide chain. These patterns greatly influence the structure, function, and biological activities of SPs. The repeating monomer units in polysaccharides such as glucose, xylose, galactose, and fucose consist of hydroxyl groups that can be sulfated. For example, sulfation in heparin and heparan sulfate occurs at C2 of iduronic acid and at N or C6 of glucosamine, while in chondroitin sulfate, sulfation occurs at the C4 and C6 positions of the galactosamine residues [18]. The sulfation position contributes to the three-dimensional conformation of the polysaccharide and also determines the interaction of polysaccharides with other molecules. For example, the differences in the sulfation patterns in fucoidan affect its anti-inflammatory and antiviral activity [19]. 

The average number of hydroxyl groups substituted by sulfate groups is referred to as the degree of substitution (DS). The value is often expressed as a ratio and provides information about the extent of sulfation. DS affects several characteristics in polysaccharides, such as viscosity, solubility, and biological properties. Excessive sulfation might cause loss of bioactivity or instability of the structure [20]. The sulfation patterns and DS dictate the biological activities and therapeutic effects of SPs. For example, DS and sulfate positionings prevent the attachment of viruses to the host cells, improving the antiviral effects of SPs. Also, the specific sulfation patterns with a high DS in Heparin affect the interaction with antithrombin III, hence improving the anticoagulant activity [21]. Increased sulfation enhances negative charge density, strengthening electrostatic interactions with positively charged domains of growth factors, cytokines, coagulation factors, and viral proteins [4]. However, biological effects depend more on the positioning of sulfate than on total sulfate content. Excessive sulfation may disrupt conformational stability and reduce selectivity [22].

### 2.3. Biological Sources of SPs

SPs have a wide range of distribution in both aquatic and terrestrial ecosystems. They can be naturally found in marine, terrestrial and microbial environments in different types.

Marine organisms play a major role as one of the most abundant and diverse producers of SPs. Among them, seaweed is popular for its SPs with many bioactivities. Seaweed, also known as macroalgae, consists of three types, namely, Green, red, and brown algae where the major types of SPs are Ulvan, Carrageenan, and fucoidans, respectively [23]. Furthermore, microalgae, marine sponges, marine mollusks, sea cucumbers, and marine bacteria are also used as sources of SPs [24]. Table 1 summarizes the types of SPs obtained from marine organisms and mammals with their monosaccharide composition and examples of their potential bioactivities.

Apart from marine sources, SPs are also found in terrestrial plants and fungal kingdoms. Even though the availability of SPs in plants is relatively rare compared to their marine counterparts, some species consist of galactans and arabinogalactans in roots, seeds, or cell wall [25]. Terrestrial fungi, such as *Aspergillus*, *Schizophyllum*, and *Ganoderma* are rich in SPs and found in their extracellular matrices or cell walls. Certain bacterial species and lichens have also been found to have SPs where they play structural roles and exhibit bioactive characteristics such as anti-viral and anti-inflammatory effects [26].

**Table 1:** Types of SPs obtained from marine organisms and mammals for comparison, and their potential bioactivities.

Source	Type of Sulfated Polysaccharide	Monosaccharide Composition	Potential Bioactivities	Examples	References
Green algae	Ulvan, sulfated arabinans, arabinogalactans	Rhamnose, Glucuronic acid, Xylose, galactose, arabinose	Antioxidant, Anticoagulant, immunomodulation, antitumor, antiviral,	*Ulvan*, *Enteromorpha*, *Monostroma*, *Codium*, *Caulerpa*, *Halimeda, Bryopsis, Chaetomorpha*, *Capsosiphon*	[9]
Red algae	Carrageenan, Agar, Porphyran, Rhamnan sulfate, sulfated galactans, sulfated glucuronogalactan	Galactose, Rhamnose, xylose, mannose	Anticoagulant, Antiviral, Anticancer, Anti-allergic, Antioxidant	*Grateloupia indica*, *Gigartina skottsbergii*, *Lomentaria catenate*, *Porphyra haitanensis*	[27]
Brown algae	Fucoidan, galactofucan, Sulfated polymannuroguluronate	Fucose, Mannuronate	Anticoagulant, Antiviral, Antioxidant, Anticancer, Immunomodulatory, Bone health, Gut health	*Sargassum thunbergii, Ecklonia cava*, *Laminaria japonica*, *Lessonia vadosa*, *Sargassum fusiforme*, *Undaria pinnatifida*	[28]
Microalgae	Sulfate containing exopolysaccharides	Xylose, Glucose, Mannose, Galactose, Fucose, Fructose	Antiviral, Antioxidant, Anti-lipidaemic, Antiglycaemic, Anti-tumor	*Cylindrotheca Closterium*, *Navicula salinarum*, *Chlorella stigmatophora*, *Tetraselmis* sp., *Isochrysis* sp., *Porphyridium* sp., *Rhodella reticulata*	[29]
Marine invertebrates	Heparin, Glycosaminoglycan, Chondroitin sulfate	Fucose, Glucuronic acid, N-acetyl galactosamine, iduronic acid	Antiviral, Used in dietary supplements,	Sea cucumber (*Stichopus japonicus*, *Ludwigothurea grisea*, *Apostichopus japonicus*), Ascidians (*Styela plicata*), Sea urchins (*Astragalus membranaceus*, *Strongylocentrotus nudus*), Nudibranchs	[30]
Mammals	Heparin/ Heparin sulfate, chondroitin sulfate, Keratan sulfate, Dermatan sulfate	2-O-sulfated iduronic acid, 6-O-sulphated, N-sulfated glucosamine, N-acetyl galactosamine and glucuronic acid	Anticoagulant, antithrombin, Anti-inflammatory, Anti-osteoarthritic,	Bovine lung, Porcine intestine, Cornea, cartilage, bone	[31]

### 2.4. Extraction, Purification, Characterization Methods

#### 2.4.1. Extraction Methods

To extract the SPs from their sources, several extraction techniques are widely used in the research and industrial fields. The choice of the extraction procedure depends on the organism type and the desired complexity of the outcome.

Hot water extraction is one of the most used extraction techniques for SPs, especially for SPs from marine algae and fungi. Often, dried biomass is boiled in water at varying temperatures ranging from 50–100 °C to dissolve water-soluble SPs. Even though this method is simple and cost-effective, there is a risk of thermal degradation of the polysaccharides [3].

Enzymatic extraction is another type of extraction that is used to extract SPs. Several specific enzymes such as Celluclast, cellulase, or pectinase are used to degrade the structural matrix to release polysaccharides without harsh conditions. This technique preserves bioactivity and minimizes structural damage, but the cost of the enzyme and the need for optimization might be potential cons [32].

In some cases, chemical treatments such as dilute acids, bases, or organic solvents like ethanol are used to extract SPs. These techniques are known to be efficient, but the harsh conditions can alter the structure of the polysaccharides which affects bioactivities. 

Another method is Ultrasound- and Microwave-assisted extraction which uses sound waves and microwaves to disrupt the cell walls and extract SPs with higher yield and reduced extraction time [33].

#### 2.4.2. Purification Methods

The crude extract of SPs is further purified to remove unnecessary compounds such as proteins, nucleic acids, and pigments. Several purification techniques are widely used. Centrifugation and filtration help to remove insoluble parts from the crude extract and most researchers use alcohol precipitation to separate polysaccharides. Ethanol or methanol in desired ratios are used in this method. Proteins are often removed using trichloroacetic acid. Chromatographic techniques such as ion exchange and size-exclusion chromatography are also popular in purifying SPs from the crude extract. Ion-exchange chromatography is used with charged resin columns like DEAE-sepharose to separate SPs according to their charge and sulfate content while size-exclusion chromatography separates SPs based on the molecular weight [34].

Further, ultrafiltration and gel electrophoresis techniques are also used to purify SPs from their crude extract.

#### 2.4.3. Characterization Methods

To study and understand the chemical structure, degree of sulfation, monosaccharide composition, and biological activities of SPs, characterization is crucial. The monosaccharide composition can be determined using High-performance Liquid Chromatography (HPLC) or Gas chromatography-mass spectrometry (GC-MS). The functional groups of the SPs compounds can be identified using Fourier Transform Infrared Spectroscopy (FTIR) [35]. It identifies functional groups such as sulfate, hydroxyl groups and carboxyl groups. Nuclear Magnetic Resonance (NMR) spectroscopy is used to obtain detailed information, including sulfate group position, linkage type, and sugar backbone. Gel permeation chromatography (GPC) or SEC coupled with Multi-Angle Light Scattering (MALS) is used to determine the molecular weight, while Scanning Electron Microscopy (SEM) visualizes surface morphology and particle size of purified SPs and X-ray diffraction (XRD) is used to determine the crystalline or amorphous nature of the polysaccharide [36].

### 2.5. Biological Implications of SPs

SPs exhibit a wide range of biological activities and have significant implications in physiological and pathological processes. Heparin and fucoidan are examples of SPs that are well known for their capacity to prevent blood clotting. They increase the activity of coagulation factors, especially antithrombin III, and inhibit the formation of clots. In therapeutic settings, this characteristic is frequently used to treat and prevent thromboembolic illnesses [37]. By suppressing the activity of pro-inflammatory enzymes like cyclooxygenase-2 (COX-2) and lowering the expression of inflammatory cytokines like (Tumor necrosis factor alpha) TNF-α and (interleukin)IL-1β, SPs modify inflammatory pathways. They may be used to treat chronic inflammatory conditions like arthritis and inflammatory bowel disease because of these effects [38]. Numerous SPs exhibit strong antiviral properties, especially those derived from marine algae. By attaching themselves to host cell surface receptors or viral envelope proteins, they obstruct viral adsorption, entrance, and reproduction. Their effectiveness against coronaviruses, HIV, and the herpes simplex virus (HSV) are a few examples [39].

On tumor cells, SPs have pro-apoptotic and antiproliferative properties. They can cause tumor cell death, alter immunological responses, and prevent angiogenesis. These characteristics are being actively studied to create new cancer treatments [40]. By stimulating natural killer (NK) cells, dendritic cells, and macrophages, these substances can improve the immune response. They are useful in increasing immunity against infections or malignancies because they also promote the synthesis of immune-modulatory cytokines. SPs lower oxidative stress, which is linked to aging and several chronic illnesses, and scavenge free radicals. Their preventive actions against diseases like neurodegeneration and cardiovascular disease are facilitated by their antioxidant capability [41]. As prebiotics, certain SPs encourage the development of healthy gut bacteria. They aid in better digestion, immunological regulation, and the avoidance of disorders linked to the gut by improving gut health [42].

## 3. Mechanism of Action of SPs in Cancer Therapy

The role of SPs in cancer therapy is versatile since they are involved in different pathways that trigger anticancer effects. Their activity directly affects cancer cells or modulates the tumor-microenvironment and improves host immune responses in many ways.

### 3.1. Induction of Apoptosis

Apoptosis, also known as programmed cell death, is a strictly regulated process crucial for removing damaged or unwanted cells. SPs are involved in this process either by intrinsic or extrinsic apoptotic pathways [43]. Through oxidative stress and regulation of mitochondrial membrane potential, SPs damage mitochondrial integrity in the intrinsic pathway. A crucial event that sets off the apoptosome complex’s assembly is the release of cytochrome c into the cytosol as a result of this disruption. After being recruited and activated by the apoptosome, caspase-9 cleaves and activates executioner caspases, including caspase-3. DNA fragmentation, chromatin condensation, and membrane blebbing are among the distinctive biochemical and morphological alterations linked to apoptosis that result from this cascade.

**Figure 1:**
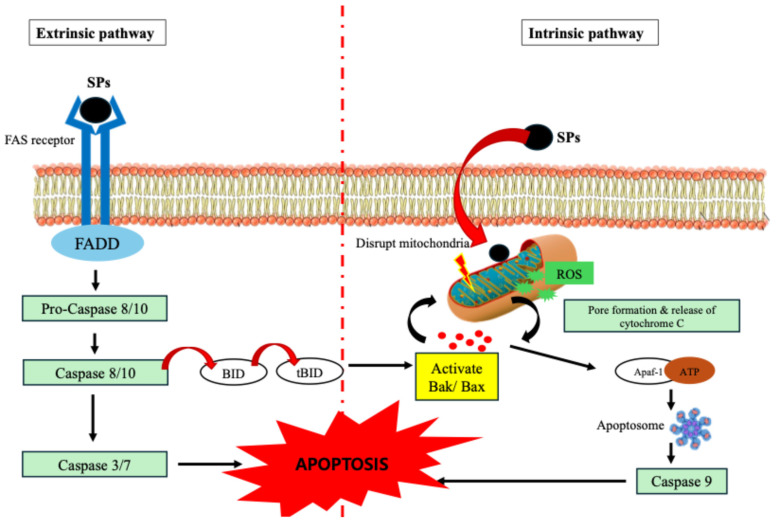
Schematic illustration of SP-induced apoptosis via both extrinsic and intrinsic pathways. In the extrinsic pathway, SPs activate the FAS receptor, leading to recruitment of FADD and sequential activation of pro-caspase-8/10 and caspase-3/7. Activated caspase-8 cleaves BID, linking to the mitochondrial pathway. In the intrinsic pathway, SPs disrupt mitochondrial integrity through ROS generation and activation of Bax/Bak, promoting cytochrome c release and apoptosome formation (Apaf-1/ATP complex), which activates caspase-9. The convergence of these cascades ultimately results in programmed cell death. Abbreviations: SPs, sulfated polysaccharides; FADD, Fas-associated death domain; BID, BH3-interacting domain death agonist; ROS, reactive oxygen species; Apaf-1, apoptotic protease activating factor-1; ATP, adenosine triphosphate.

By upregulating or activating cell surface death receptor pathways and associated adaptors like the TNF receptor or FAS (CD95), SPs can also trigger the extrinsic route. This connection facilitates the activation of initiator caspase-8 and the recruitment of adaptor proteins, such as FADD. By cleaving the Bcl-2 family protein BID, which connects the extrinsic and intrinsic pathways, Caspase-8 can either directly activate executioner caspases or enhance the apoptotic signal [40]. The balance of pro-apoptotic and anti-apoptotic Bcl-2 family proteins is known to be impacted by SPs. They inhibit cytochrome c release by downregulating the expression of anti-apoptotic proteins such as Bcl-2 and Bcl-xL, which typically stabilize the mitochondrial membrane. At the same time, pro-apoptotic proteins like Bak and Bax, which create holes in the mitochondrial membrane and promote the release of cytochrome c, are elevated by SPs [40]. However, due to their macromolecular nature, SPs are not presumed to freely diffuse across the plasma membrane. Instead, their pro-apoptotic effects are primarily mediated through interactions with cell surface receptors, which initiate intracellular signaling cascades that converge on mitochondrial dysfunction and intrinsic apoptosis [44]. This dual modulation increases the demise of cancer cells by tipping the scales in favor of apoptosis. SPs are attractive candidates for anticancer treatments because these mechanisms allow them to efficiently target cancer cells while preserving healthy cells. Figure 1 depicts both the extrinsic and intrinsic apoptosis of tumor cells.

### 3.2. Immunomodulation to Enhance Anti-Tumor Immunity

SPs are essential for immune system modulation that improves anti-tumor immunity. They accomplish this by interacting with different immune system components, creating an atmosphere that supports strong immune monitoring and the removal of tumors [44]. SPs are capable of activating macrophages and dendritic cells which are essential antigen-presenting cells responsible for commencing adaptive immune responses. Through pattern recognition receptors (PRRs) such as Toll-like receptors (TLRs), especially TLR4, SPs activate macrophages [45]. Tumoricidal activity depends on the production of pro-inflammatory cytokines (e.g., TNF-α, IL-6), reactive oxygen species (ROS), and nitric oxide (NO) by macrophages as a result of this activation. To boost the immune response, activated macrophages also phagocytose tumor cells and expose T-cells to tumor antigens [46]. Major Histocompatibility Complex (MHC) molecules are used by SPs to help DCs process and present tumor antigens by promoting their maturation and activation. In lymph nodes, mature DCs stimulate naïve T-cells, especially cytotoxic T lymphocytes (CTLs), to target tumor cells preferentially [45,47].

Also, SPs enhance the cytotoxic ability of NK cells, which directly destroy the tumor cells without prior sensitization [48]. NKG2D and other activating receptors on NK cells that detect stress-induced ligands on tumor cells are upregulated by SPs. They increase the synthesis of the chemicals granzyme and perforin, which cause target cells to undergo apoptosis. Additionally, SPs may increase the sensitivity and effectiveness of NK cells against malignancies by decreasing the production of inhibitory signals on tumor cells [49].

SPs alter the cytokine profile within the tumor microenvironment (TME), promoting a shift from an immunosuppressive to an immunostimulatory state. SPs increase the synthesis of cytokines, including TNF-α and interferon-gamma (IFN-γ). Activated T-cells and NK cells release IFN-γ, which is essential for increasing antigen presentation by APCs and encouraging tumor cell death [50]. TNF-α attracts more immune cells to the TME and damages the tumor vasculature. Immunosuppressive cytokines, including interleukin-10 (IL-10) and transforming growth factor-beta (TGF-β), which are frequently increased in tumors and decrease anti-tumor immunity, are inhibited by SPs. SPs help immune cells, including T-cells and NK cells, operate against by reducing these factors [51].

### 3.3. Inhibition of Tumor Invasion and Metastasis

When SPs target important molecular and cellular mechanisms involved in tumor dissemination, they effectively prevent cancer cell invasion and metastasis [52]. The multi-step process of metastasis includes adhesion, intravasation, extravasation, colonization at secondary locations, migration of cancer cells, and destruction of the extracellular matrix (ECM) [53]. SPs impede the progression of tumors by interfering with multiple of these processes. Proteolytic enzymes called MMPs, including MMP-2 and MMP-9, and urokinase plasminogen activator (uPA) break down elements of the basement membrane and extracellular matrix, which promotes tumor invasion and metastasis. By modifying signaling pathways like MAPK, NF-κB, and PI3K/Akt, all of which are frequently elevated in cancer cells, SPs suppress the production and enzymatic activity of MMPs [54]. SPs stop the ECM from breaking down, which is a crucial obstacle to tumor cell migration, by lowering MMP activity. Likewise, SPs suppress uPA, which changes plasminogen into plasmin, a protease that triggers MMPs and encourages the breakdown of extracellular matrix. In addition to stopping local invasion, this dual inhibition of proteolytic enzymes also prevents cancer cells from entering lymphatic and blood arteries [55].

Because it promotes both intravasation and extravasation during tumor spread, cancer cell adhesion to endothelial cells and adjacent tissues is an essential stage in metastasis. SPs disrupt cancer cells’ adhesion to the extracellular matrix and decrease their motility by interfering with integrin-mediated signaling pathways. Further restricting invasion, this inhibition also lessens the contact between stromal components and cancer cells. SPs decrease circulating tumor cells’ (CTCs’) capacity to stick to endothelial cells, which is a need for extravasation into secondary locations, by inhibiting selectin-mediated adhesion. Because selectins help cancer cells dock in distant organs during hematogenous metastasis, this activity is very pertinent [56].

The epithelial–mesenchymal transition (EMT), which gives epithelial cancer cells mesenchymal characteristics such increased motility and invasiveness, may be inhibited by SPs. SPs increase the expression of epithelial markers like E-cadherin while downregulating important EMT indicators like N-cadherin, vimentin, and Snail. SPs decrease the flexibility of cancer cells by reversing EMT, which hinders their capacity to invade and spread [57]. By interfering with angiogenesis, the process by which new blood vessels grow to deliver oxygen and nutrients to tumors and promote metastasis, SPs indirectly prevent tumor invasion. 

### 3.4. Interaction with Growth Factor Signaling

**Figure 2:**
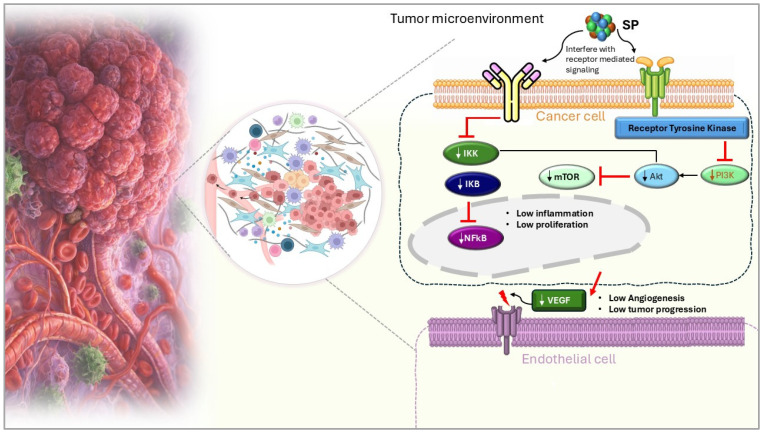
Sulfated polysaccharides (SP) modulate key signaling pathways such as PI3K/Akt/mTOR, NFkB, and VEGF-mediated angiogenesis within the tumor microenvironment [1].

By interfering with important growth factor signaling pathways, especially those involving fibroblast growth factors (FGFs) and vascular endothelial growth factor (VEGF), SPs effectively prevent tumor growth and metastasis [52]. The migration, angiogenesis, and proliferation of cancer cells, all of which aid in the development of tumors require these growth factors. By attaching themselves directly to FGFs and blocking their ability to interact with tumor cell fibroblast growth factor receptors (FGFRs), SPs disrupt FGF signaling [58]. The downstream signaling pathways, including PI3K/Akt and MAPK/ERK, which are essential for tumor cell migration and proliferation, are interfered with by this inhibition [59]. Likewise, SPs influence VEGF signaling, a major angiogenesis-promoting factor. By attaching itself to endothelial cells’ VEGF receptors (VEGFRs), VEGF promotes the development of new blood vessels (Figure 2). By directly interacting with VEGF or VEGFRs, SPs stop this binding and stop the activation of angiogenic pathways. The tumor’s capacity to establish the blood supply required for long-term growth and the dissemination of cancer cells to other organs is hampered by this obstruction [60].

Sulfated polysaccharides from red, green, and brown seaweeds have been extensively studied for their potential anticancer properties. Their efficacy and mechanisms vary depending on their structural composition, degree of sulfation, and the cellular targets they interact with. A direct comparison of these SPs is demonstrated in Table 2.

## 4. Biomedical Applications of Major SPs-Fucoidan

### 4.1. Fucoidan in Combination with Cancer Therapies

Chemotherapy-induced immunosuppression often leads to an increased risk of infections and a significant decline in patient quality of life due to a weakened immune system. This suppression mainly results from the destruction of rapidly dividing cells, including hematopoietic stem cells and immune cells like neutrophils, which are essential for fighting infections. Therefore, it is crucial to support the recovery of the immune system following chemotherapy and radiotherapy.

The SP, fucoidan, has been reported to exhibit protective effects against tumor development. Its immunomodulatory activity was evaluated in a study involving rats with dimethyl benz[a]anthracene (DMBA)-induced mammary carcinogenesis. Rats were administered fucoidan orally at 200 and 400 mg/kg body weight. The groups treated with fucoidan demonstrated a lower tumor incidence rate, reduced tumor weight, and extended tumor latency compared to the control group. Additionally, fucoidan treatment increased the levels of IL-6, IL-12, and interferon-γ. These findings suggest that fucoidan has the potential to protect against the development of mammary carcinoma [67].

Fucoidan has been reported for its utilization as a supplement with chemotherapeutic agents and radiation to treat cancer. It is believed that fucoidan either enhances the anticancer activity of the drug or the patient’s tolerance to side effects. In a study conducted by Ikeguchi et al. (2011), patients who were administered fucoidan were able to tolerate chemotherapy for longer periods without fatigue than patients who were not administered fucoidan [68]. Moreover, they found that the survival of patients with fucoidan treatment was longer than without fucoidan. Another study investigating lung cancer in vivo and in vitro highlighted the potential of fucoidan to reduce the expression of transforming growth factor-beta (TGFβ) receptors, which led to a decrease in cancer cell proliferation and progression [69]. Atashrazm et al. (2016) investigated fucoidan administered with arsenic trioxide and all trans retinoic acid as an adjuvant therapy to decrease the toxicity in patients with acute promyelocytic leukemia [70]. They identified that fucoidan exerts a synergistic effect with acute promyelocytic leukemia therapies all -trans-retinoic acid and arsenic trioxide. As the study reported, fucoidan combination with the drugs increased the apoptosis of NB4 cells and enhanced myeloid differentiation. Moreover, in vivo results provided that fucoidan and drug combination resulted in decreased tumor growth and decreased tumor cell migration [70]. In a study conducted by Russian scientists on *Fucus evanescens*, fucoidan was found to increase cancer susceptibility to radiation. It was due to the radiosensitizing activity of fucoidan and the increased inhibiting effect of X-ray radiation on the proliferation and colony formation of cancer cells [71]. Numerous studies have proved that fucoidan exerts stress on a number of cellular pathways in cancer cells and thereby increases the cancer cell susceptibility to radiation and other agents. Conversely, fucoidan confers radiation protection activity of normal cells through antioxidant and DNA protection, which helps in preventing healthy cell damage and subsequent fibrosis in healthy tissues [72].

Previous studies have demonstrated that ingestion of fucoidan extract can lead to an increase in circulating stem cells. These stem cells play a pivotal role in regenerating the hematopoietic system, including neutrophils and other immune cells. Fucoidan’s ability to enhance stem cell mobilization highlights its potential as an adjunct therapy to accelerate immune recovery post-chemotherapy [73]. Skeletal muscle atrophy is another representative feature of cancer cachexia, and is often observed in cancer patients undergoing chemotherapy. Skeletal muscle atrophy leads to a decrease in quality of life owing to a reduction in social activity and exercise, along with clinical problems, including poor tolerance to cancer therapy [74]. Fucoidan was reported to inhibit tumor- and chemotherapy-induced skeletal muscle atrophy in bladder cancer-bearing mice. In a study involving orthotopic mice transplanted with T24 cancer cells and treated with a combination chemotherapy regimen of cisplatin (CDDP) and gemcitabine (GEM), low molecular weight fucoidan (LMWF) derived from *Sargassum hemiphyllum* (average molecular weight 760 Da) significantly suppressed muscle atrophy.

The protective effects of LMWF were attributed to its ability to regulate inflammation, inhibit muscle proteolysis, and enhance protein synthesis. It effectively controlled inflammation, a major driver of muscle wasting, and suppressed muscle protein degradation by downregulating the myostatin/activin A signaling pathway and FoxO3 transcription factor activity. Furthermore, LMWF improved muscle protein synthesis through the modulation of the IGF-1 signaling axis and reduced the expression of key muscle atrophy-related genes, including MAFbx and MuRF-1. Additionally, it inhibited NF-κB activation, a pathway associated with inflammation and catabolic responses. These findings suggest that LMWF mitigates chemotherapy-induced toxicity and muscle wasting by targeting multiple pathways, highlighting its potential as an adjunct therapy to preserve skeletal muscle health during cancer treatment.

### 4.2. Fucoidan Nanoparticles as a Drug Delivery System

Many clinical studies reported the therapeutic potential of fucoidan for the treatment of cancer through oral administration. However, researchers consistently noted that this method resulted in low bioavailability and high clearance rates of fucoidan, which possess significant challenges to their effectiveness as antitumor drugs. To address this limitation, a promising strategy involving the development of fucoidan-based nanoparticles was introduced. These can be engineered with specific characteristics that allow for adjustments in pharmacokinetics and enhance the accumulation of fucoidan derivatives in tumors through enhancing the binding with P-selectins [75]. Fucoidan is currently used in conventional drug encapsulation and can be used for oral intake or intravenous therapies [23]. Seaweed polysaccharides have attracted significant interest in this field due to their numerous advantages, including ease of extraction and purification, cost-effectiveness, non-toxicity, biodegradability, biocompatibility, and versatility in application [76]. Additionally, these natural hydrophilic polysaccharides offer a practical solution for encapsulating hydrophobic anticancer agents, helping to reduce side effects and minimize the uncontrolled dispersion of chemotherapy drugs within the body, thereby enhancing their therapeutic efficiency and overall efficacy.

Most fucoidan-based systems have been primarily developed for the treatment of breast cancer. One notable approach involved the electrostatic assembly of fucoidan with positively charged polyethyleneimine, resulting in doxorubicin (DOX)-loaded nanoparticle systems, as described by Pawar et al. (2018) [77]. These varied in size from 41 to 160 nm, with larger sizes corresponding to higher fucoidan ratios, and exhibited a polydispersity index (PDI) of 0.153 along with a negative charge. Drug release studies indicated that approximately 30% of DOX was released within the first 24 h across different pH levels. These NPs effectively arrested cell cycle progression at the G1-S phase, leading to apoptosis. Both in vitro and in vivo studies demonstrated enhanced cytotoxicity and tumor shrinkage compared to the free drug. In another study, Lu et al. (2017) encapsulated DOX into protamine/fucoidan nanoparticles [78]. Stable nanoparticles were produced only at weight ratios below 1.0, resulting in negatively charged nanoparticles with an average size of approximately 180 nm. At pH 7.4, around 70% of DOX was released within 24 hours, while at pH 4.5, the drug release was significantly accelerated, with 80% released within 12 h. This rapid release was attributed to the deionization ofchemical groups and weakened electrostatic interactions between the NPs and DOX. These exhibited higher cytotoxicity against MDA-MB-231 human breast cancer cells compared to the free drug, likely due to differences in cellular uptake mechanisms [78].

Additionally, Manivasagan et al. (2016) developed gold nanoparticles coated with fucoidan and loaded with DOX [79]. These remained stable for up to six months and demonstrated a faster drug release at physiological pH, potentially reducing toxicity to normal tissues [79]. The IC50 of these nanoparticles was 5 μg/mL, compared to 30 μg/mL for free DOX, further confirming the enhanced efficacy of this system against MDA-MB-231 breast cancer cells [79]. Fucoidan-based nanoparticles can be a platform for creating a site-directed delivery system for hydrophobic compounds. For this purpose, the nanoparticles are constructed from chitosan and fucoidan. These type of nanoparticles increase the solubility of the chemotherapy drugs and their blood circulation while increasing the accumulation in the tumors [80]. These chitosan/fucoidan nanoparticles are also used to reduce the toxicity of antitumor drugs while improving their effectiveness. 

Another approach for delivering hydrophobic substances to tumors involves the use of chitosan-fucoidan nanoparticles, specifically with Piperlongumine (PL). PL is a novel pro-oxidant drug that induces cancer-specific apoptosis by increasing intracellular ROS. Despite its potential, PL has poor solubility and limited clinical application. However, chitosan-fucoidan nanoparticles (CS-FNP) have been shown to effectively encapsulate PL, enhancing its solubility in water and improving its bioavailability. Studies have demonstrated that PL-loaded CS-FNP nanoparticles (PL-CS-FNP) exhibit greater cytotoxicity against PC-3 prostate cancer cells compared to free PL. Additionally, these nanoparticles display selective anticancer activity, showing significantly higher cytotoxicity against PC-3 tumor cells than against non-transformed human dermal fibroblast (hDFB) cells [81]. It is important to note, however, that the effectiveness of chitosan-fucoidan nanoparticles for delivering antitumor drugs may be limited at pH levels above 6.5. This is due to the deprotonation of chitosan, which can lead to decreased solubility in water, potentially affecting the delivery and efficacy of the encapsulated drugs [82]. Table 3 is organized based on nanoparticle formulation to allow comparison of delivery systems, preparation methods, and drug combinations across studies. The inclusion of cancer type within the table still enables readers to identify disease-specific applications.

## 5. Clinical Research of Fucoidan

In recent years, there have been few investigations on the possible systemic effects of oral fucoidan, both locally and internationally, with the majority being undertaken in vitro or in animal models, mainly mice. The lack of clinical studies may be linked to numerous issues. For starters, the molecular structure of fucoidan is extremely complicated and diverse, making it difficult to ensure the correctness and representativeness of study results. Furthermore, fucoidan absorption after oral administration is quite poor, and its concentration inside the body is difficult to correctly detect, complicating its clinical assessment [91]. Extensive research on the anti-tumor effects and associated processes of fucoidan has revealed its low toxicity and anti-inflammatory capabilities, positioning it as a viable adjuvant therapy for cancer patients undergoing conventional treatment. Myers et al. (2016) conducted a 12-week, double-blind, controlled trial on individuals with osteoarthritis at random [92]. The efficacy of the medication was determined by a thorough osteoarthritis test, while the safety was determined by assessing liver function, cholesterol, hematological function, renal function, and closely monitoring side events. The results indicated that taking 300 mg of fucoidan is safe and well-tolerated in people. However, fucoidan had no significant benefit in treating OA symptoms compared to placebo [92].

In a clinical study conducted in Japan, 13 patients with HTLV-1 associated myelopathy/tropical spastic paralysis (HAM/TSP) were enrolled and administered 6 g of fucoidan daily for at least 6 months. The results showed a significant decrease in pre-viral DNA load, with a reduction of about 42.4% compared to the control group [93]. This study provided the first evidence of the anti-inflammatory effects of fucoidan in patients with advanced cancer. In a prospective open-label clinical trial involving 20 patients with advanced cancer, participants took 4 g of oral fucoidan daily for a minimum of 4 weeks. The findings indicated that major pro-inflammatory cytokines, such as interleukin-1β (IL-1β), IL-6, and TNF-α, significantly decreased after two weeks of continuous fucoidan consumption. However, the quality of life scores, including measures of fatigue, did not show significant improvement during the study period [94]. Low-molecular-weight fucoidan (LMWF) is commonly used as a food supplement in cancer patients. Hsiang et al. conducted a prospective, randomized, double-blind, controlled trial to assess the efficacy of LMWF as a complementary therapy to chemotherapy and targeted drugs in patients with metastatic colorectal cancer (mCRC). The study involved 54 patients, with 28 patients in the experimental group receiving 4 g of LMWF daily, while 26 patients in the control group took 4 g of cellulose daily. The results showed a significant difference in disease control rate (DCR) between the experimental and control groups, with DCRs of 92.8% and 69.2%, respectively. This study is the first clinical trial to evaluate the efficacy of LMWF as an adjunct treatment in mCRC patients, demonstrating that LMWF combined with chemotherapy and targeted drugs can significantly improve the DCR [95].

A study investigated the efficacy of fucoidan in reducing the adverse effects of cisplatin treatment in 24 patients with unresectable advanced gastric cancer. The patients were randomly assigned to either the fucoidan-treated group (*n* = 12) or the control group (*n* = 12), with all patients receiving cisplatin. The fucoidan group took 150 mL/day of a liquid containing 4.05 g of high-molecular-weight fucoidan from *Cladosiphon okamuranus* for six months following chemotherapy. The results showed that fucoidan suppressed the occurrence of diarrhea, reduced general fatigue, and helped maintain a favorable nutritional status, as indicated by a higher prognostic nutritional index (PNI: 47.6 ± 6.1 vs. 39.4 ± 8.2; *p* = 0.028). The fucoidan group was able to continue chemotherapy for a longer period (7.4 months vs. 4.6 months; *p* = 0.044), and the mean survival time was significantly better in the fucoidan group compared to the control group (12 months vs. 8 months; *p* = 0.039). These findings suggest that fucoidan may help mitigate the adverse effects of cisplatin and improve the survival and quality of life of cancer patients undergoing chemotherapy [96].

In 2022, enrollment began for two clinical studies looking into the advantages of fucoidan dietary supplements in cancer patients. A randomized parallel research will look at how oligo-fucoidan might help reduce lung damage caused by radiation treatment in lung cancer patients. The other randomized-parallel research will look at oligo-fucoidan’s capacity to treat cachexia or sarcopenia in cancer patients. The data gathering for the two clinical studies is planned to be finished by 2024. The findings might be critical in clarifying the benefits of fucoidans in minimizing adverse effects during radiochemotherapy and promoting their use as a supplemental treatment in cancer.

A study investigated the potential pharmacokinetic interactions between fucoidan and conventional anticancer therapies, focusing on the effect of co-administering *Undaria pinnatifida*-derived fucoidan with letrozole or tamoxifen in breast cancer patients. In this open-label, non-crossover study, 20 female patients were enrolled: 10 received letrozole and 10 received tamoxifen. Each patient took 500 mg of fucoidan twice daily for 3 weeks [97]. HPLC analysis conducted at baseline and after 3 weeks revealed that fucoidan co-administration did not alter the steady-state plasma concentrations of letrozole, tamoxifen, or tamoxifen metabolites. Additionally, no adverse effects of fucoidan were reported during the study period.

The results suggest that fucoidan does not interfere with the pharmacokinetics of letrozole or tamoxifen. However, future well-designed randomized control trials are needed to further assess the potential pharmacokinetic interactions and clinical benefits of combining fucoidan with various anticancer drugs.

This available evidence suggests that SPs such as fucoidan supplementation combined with conventional adjuvant chemotherapy help prolong survival, reduce chemotherapy-related side effects and improve inflammation in patients with advanced cancer or metastasis.

## 6. Challenges in Utilizing SPs as Anticancer Drugs

The struggle for existence leads organisms to evolve novel adaptations such as speed, strength, and the development of chemical poisons. These toxins have extremely powerful biological activity and distinct methods of action, making them ideal candidates for therapeutic study. SPs have been praised for their bioactivity in extremely diluted circumstances. Despite significant research into their anticancer potential and targeting capabilities, their usage as a cancer therapeutic molecule or drug delivery system has encountered certain challenges. Pure fucoidan is indeed a costly and limited biomolecule as it is a natural polymer obtained from brown seaweeds. Obtaining adequate quantities of these chemicals for future research remains challenging. Many researchers prefer to use crude fucoidan, which complicates the investigation of structure-bioactivity relationships. The processes of extraction and purification are complex and involve multiple meticulous steps [98]. Commercially available fucoidans, such as those derived from *F. vesiculosus*, *M. pyrifera*, *L. japonica*, and *U. pinnatifida*, are widely marketed but come with high production costs. This financial barrier likely limits their use in research applications and hinders their broader adoption in industrial settings. The structural characteristics of purified fucoidan often differ from those of crude forms. However, in some cases, the purified form closely resembles native fucoidan, as seen with crude *F. vesiculosus* fucoidan, where purification primarily reduces the total phenolic content while maintaining its fundamental structural integrity [99]. Comparing the activity of fucoidan from various species in crude and purified forms presents substantial hurdles. Only pure forms of fucoidan from *Ecklonia cava*, *Costaria costata*, and *Sargassum horneri*, for example, have anticancer activity against human skin melanoma (SK-MEL-28) and human colon cancer (DLD-1) cell lines [100]. In contrast, the antiproliferative and antioxidant properties of crude fucoidan isolated from *F. vesiculosus* may be due to phenolic pollutants. This is corroborated by the fact that the purified form of *F. vesiculosus* fucoidan does not display similar effects, emphasizing the role of contaminants in crude preparations in determining bioactivity [99].

Another challenge is the structural complexity. The monosaccharide content and structural characteristics from different seaweed species and harvest seasons were investigated by many researchers. Its unusual structure makes manipulation and chemical synthesis extremely difficult. These challenges have also hampered the creation of low-cost synthetic copies of fucoidan, limiting its use and accessibility [101]. Fucoidan’s complicated structure and the limited number of research on pure fucoidan make it difficult to determine structure-bioactivity connections. However, the anticancer impact of L-fucose alone has not been shown to be substantial in the human breast cancer cell line MCF-7. This shows that fucoidan’s bioactivity may be regulated by its whole structure rather than specific components such as L-fucose [102].

The absorption efficiency of SPs varies greatly depending on their structural properties, including molecular weight, degree of sulfation, and monosaccharide content. These structural changes alter their bioavailability and absorption in certain organs, which affects their overall therapeutic potential and biological activity [103]. The dose of fucoidan for in vitro studies mostly resides in the range of µg/mL due to its highly branched nature. Safety analysis and biodegradability testing of fucoidan are ongoing. Fucoidan has long been consumed as a food ingredient in Asian diets. Despite its established use as a food and dietary supplement, recent studies have focused on assessing its safety, including evaluating hepatotoxicity and nephrotoxicity for both pure extracts and low-molecular-weight (LMW) fucoidan. Acute toxicity trials conducted in vivo over four weeks have shown no significant adverse effects. Additionally, in murine models, fucoidan derived from *F. vesiculosus*, *L. japonica*, and *U. pinnatifida* exhibited no toxicological manifestations [104,105]. Although considered safe, fucoidan is not a biodegradable polymer and is believed to be excreted intact in urine. Moving forward, researchers need to reach a consensus on the classification of medications that can be developed from marine algae, elucidate their mechanisms of action, and explore the potential of these phytochemical compounds as dietary supplements, particularly for cancer patients [106]. Fucoidan’s safety profile, particularly when utilized as nanoparticles or a coating material for different nano systems, has yet to be thoroughly established. More research is needed to establish fucoidan nanoparticles’ potential as a safe and effective anticancer therapy, as well as their long-term safety and biocompatibility.

Recent research emphasizes the necessity to test fucoidan in prospective clinical trials, particularly in conjunction with chemotherapy, in order to improve cancer patient outcomes. Preclinical studies have looked at the possible hepatic metabolism-mediated interactions between fucoidan and chemotherapy medicines. Fucoidan produced from *F. vesiculosus* displayed strong synergistic benefits when paired with paclitaxel and tamoxifen [107], additive efficacy with topotecan, and no antagonistic action with letrozole [108]. These data indicate that *F. vesiculosus* fucoidan may have few drug-supplement interactions with the CYP450 or COMT hepatic metabolic pathways. Furthermore, fucoidan does not stimulate tumor growth; its immunomodulatory effects may perhaps aid in tumor suppression. However, these observations must be validated by thorough clinical investigations. Future study should focus on proving the efficacy and safety of fucoidan when utilized.

## 7. Conclusions and Future Perspectives

The current landscape of cancer therapy has increasingly recognized SPs as a promising source for the discovery of bioactive molecules with diverse chemotherapeutic effects across various malignancies. Recent data indicates that over half of the FDA-approved medications in recent years have either been directly derived from marine sources or developed using synthetic analogs of these natural compounds [40]. This trend underscores the potential of marine-derived SPs, which exhibit enhanced bioavailability, a diverse chemical profile, and non-reductant cytotoxicity, making them attractive candidates for cancer treatment.

SPs extracted from seaweeds, such as fucoidan and carrageenan, have emerged as potential lead pharmacophores due to their multifaceted therapeutic properties. These compounds not only demonstrate anticancer activity but also possess immunomodulatory, anti-inflammatory, and antioxidant effects, which can contribute to their efficacy in cancer therapy. However, several challenges hinder their pharmaceutical application, primarily related to their bioavailability, the need for improved separation techniques, the purity of the isolates, and the specificity of their action. The complexity of cancer biology necessitates that these drugs exhibit multi-target specificity, tailored to the unique cellular and tissue contexts of different malignancies. Moreover, SPs serve as druggable mediators due to their extensive range of therapeutic interventions, cost-effective commercial production, and promising results from pre-clinical and clinical studies. The optimism surrounding the commercialization of these compounds is bolstered by the feasibility of extensive on-site and off-site harvesting of marine organisms, coupled with low-cost cultivation practices. This accessibility facilitates the large-scale production of SPs, which is further enhanced by advancements in chemical synthesis techniques that allow for the efficient manufacture of these compounds.

The future of cancer therapy may be significantly influenced by the innovative isolation and screening of SPs from seaweeds, positioning them as novel pharmacological agents against various cancers. The development of sulfated polysaccharide-based nanoparticles represents a particularly exciting avenue, as these nanoparticles can provide sustained drug release, high stability, and biocompatibility, which are crucial for their application in clinical trials. By incorporating targeting moieties into these nanoparticles, researchers can enhance their therapeutic efficacy while minimizing undesirable side effects, thereby improving patient outcomes.

Furthermore, the creation of such drug candidates has the potential to augment existing therapies, paving the way for advancements in personalized and precision medicine. As research continues to unveil the mechanisms of action and therapeutic potential of SPs, their role in cancer treatment is likely to expand, offering new hope for patients facing various malignancies. The integration of these natural compounds into modern therapeutic regimens could revolutionize cancer care, making it more effective and tailored to individual patient needs. 

## Figures and Tables

**Table 2:** Comparative analysis of Anticancer mechanisms, Efficacy, and Applications of Major Seaweed-Derived SPs.

Sulfated Polysaccharide	Primary Anticancer Mechanism	Efficacy (In Vitro/In Vivo)	Potential Applications	Examples	References
Fucoidan (from brown algae)	Induces apoptosis via mitochondrial and death receptor pathways; inhibits angiogenesis, metastasis, and tumor cell proliferation; modulates immune responses and gut microbiota	Demonstrates strong cytotoxicity and growth inhibition in various cancer cell lines (colon, breast, liver, and lung); proven in multiple animal models; some clinical evidence available	Widely explored for drug delivery systems, immunotherapy adjuvants, and combination chemotherapy; progressing toward clinical translation	*Fucus vesiculosus*, *Sargassum hemiphyllum*, *Cladosiphon okamuranus*, *Sargassum fusiforme*, *Kjellmaniella crassifolia*, *Turbinaria conoides*, *Saccharina latissima*	[61,62]
Carrageenan (Red algae)	Induces apoptosis and cell cycle arrest; shows immunostimulatory and anti-proliferative effects; may activate inflammatory pathways at high concentrations	Moderate cytotoxicity; effectiveness depends on sulfate type (κ-, ι-, or λ-carrageenan); limited in vivo and clinical validation	Promising as a drug carrier, hydrogel, or nanocomposite for targeted therapy; potential adjuvant role in delivery formulations	*Kappaphycus striatum*, *Chondrus armatus*	[63,64]
Ulvan (from green algae)	Exhibits antioxidant and immunomodulatory actions; suppresses tumor growth through ROS scavenging and inhibition of NF-κB and MAPK pathways	Mild to moderate anticancer activity; strong synergistic effects when combined with chemotherapeutics or nanoparticles; limited mechanistic data	Useful in biocompatible scaffolds and nanoformulations; promising antioxidant-based cancer-preventive applications	*Ulva lactuca*, *Ulva intestinalis*, *Ulva ohnoi*, *Ulva rigida*	[65,66]

**Table 3:** Fucoidan-based nanoparticles prepared for anticancer therapies.

Species	System	Drug	Preparation Method	Cancer Type	Reference
*Fucus vesiculosus*	Fucoidan/ polyethylenimine	DOX	Polyelectrolyte complexation	Breast cancer	[77]
*Fucus vesiculosus*	Rutin/fucoidan complex	Rutin	Fucoidan was added dropwise to rutin solution	Cervical cancer	[83]
*Fucus vesiculosus and shrimp shell*	Silver nanoparticles with chitosan/fucoidan coating		Silver nitrate was added to fucoidan solution, followed by the addition of fucoidan	Cervical cancer	[84]
*Fucus vesiculosus*	Fucoidan-coated copper sulfide NPs (F-CuS)		Synthesis of sodium citrate-stabilized copper sulfide NPs (CuS) and coating using the layer-by-layer (LbL) technique (PAH and Fu)	Cervical cancer	[85]
*Fucus vesiculosus*	Fucoidan capped gold nanoparticles	DOX	Addition of fucoidan to Gold (III) chloride trihydrate, under magnetic heater stirrer	Breast cancer	[79]
*Saccharina cichorioides*	Fucoidan-coated gold nanoparticles		Synthesis of Fucoidan-mimetic (FM)-glycopolymers via a free-radical chain transfer polymerization reaction	Colon cancer	[86]
*Fucus vesiculosus*	Fucoidan-drug based nanoparticles	PAX. DOX, MEK	Co-encapsulation and layer-by-layer assembly	Colon cancer	[87]
*Fucus vesiculosus, Macrocystis pyrifera, Undaria pinnatifida*	Dextran-coated superparamagnetic iron oxide nanoparticles (SPIONs) with fucoidan		Coating of SPIONs with fucoidan	Glioma cancer	[75]
*Fucus vesiculosus,*	Gold nanoparticles coated with fucoidan	DOX	Fucoidan poured into gold chloride hydrate solution	Eye cancer	[88]
*Fucus vesicuosus*	Fucoidan drug based nanoparticles	DOX, PAX	Dextran-coated superparamagnetic iron oxide NPs (SPIONs) with fucoidan	Melanoma	[87]
*Fucus vesicuosus*	Fucoidan-coated copper sulfide		Synthesis of sodium citrate-stabilized copper sulfide nanoparticles and coating using the layer-by-layer method	Lung cancer	[89]
*C. okamuranus*	Liposome Encapsulating Fucoidan		Mechanochemical method	Osteosarcoma	[90]
*L. japonica*	Fucoidan/ protamine	DOX	Self-assembled colloidal nanocomplex formed by electrostatic interactions.	Breast cancer	[78]

## Data Availability

Dataset available on request from the authors.
